# Single-cell adhesion strength and contact density drops in the M phase of cancer cells

**DOI:** 10.1038/s41598-021-97734-1

**Published:** 2021-09-16

**Authors:** Rita Ungai-Salánki, Eleonóra Haty, Tamás Gerecsei, Barbara Francz, Bálint Béres, Milán Sztilkovics, Inna Székács, Bálint Szabó, Robert Horvath

**Affiliations:** 1 Department of Biological Physics, ELTE Eötvös Loránd University, Budapest, Hungary; 2 CellSorter Scientific Company for Innovations, Erdőalja út 174, 1037 Budapest, Hungary; 3 Nanobiosensorics Laboratory, ELKH, Institute for Technical Physics and Materials Science, Centre for Energy Research, Budapest, Hungary

**Keywords:** Motility, Biomedical engineering, Biological techniques, Biophysics, Cancer, Medical research, Oncology, Applied optics

## Abstract

The high throughput, cost effective and sensitive quantification of cell adhesion strength at the single-cell level is still a challenging task. The adhesion force between tissue cells and their environment is crucial in all multicellular organisms. Integrins transmit force between the intracellular cytoskeleton and the extracellular matrix. This force is not only a mechanical interaction but a way of signal transduction as well. For instance, adhesion-dependent cells switch to an apoptotic mode in the lack of adhesion forces. Adhesion of tumor cells is a potential therapeutic target, as it is actively modulated during tissue invasion and cell release to the bloodstream resulting in metastasis. We investigated the integrin-mediated adhesion between cancer cells and their RGD (Arg-Gly-Asp) motif displaying biomimetic substratum using the HeLa cell line transfected by the Fucci fluorescent cell cycle reporter construct. We employed a computer-controlled micropipette and a high spatial resolution label-free resonant waveguide grating-based optical sensor calibrated to adhesion force and energy at the single-cell level. We found that the overall adhesion strength of single cancer cells is approximately constant in all phases except the mitotic (M) phase with a significantly lower adhesion. Single-cell evanescent field based biosensor measurements revealed that at the mitotic phase the cell material mass per unit area inside the cell-substratum contact zone is significantly less, too. Importantly, the weaker mitotic adhesion is not simply a direct consequence of the measured smaller contact area. Our results highlight these differences in the mitotic reticular adhesions and confirm that cell adhesion is a promising target of selective cancer drugs as the vast majority of normal, differentiated tissue cells do not enter the M phase and do not divide.

## Introduction

**Cell adhesion**^1^ is a fundamental and complex biological process of a cell anchoring to another cell or to the extracellular matrix (ECM). This process is mediated by cell surface receptor molecules, such as integrins, cadherins, selectins, and members of the immunoglobulin superfamily^2^. Cell adhesion is known to be closely related to the actin cytoskeleton, the organization of which is crucial in determining the structural and mechanical properties of living cells. Dynamically controlled cell adhesion plays a cardinal role both on the level of individual cells in intracellular signaling, migration, proliferation, differentiation, and gene expression and on the level of multicellular organisms in cell–cell communication, the developing embryo^2^, the immune system^3^, and the metastasis of tumors^4–6^. Adhesion forces act as signaling pathways. The rigidity of the ECM affects the differentiation and morphogenesis of cells. The rigidity is sensed primarily through integrin-mediated cell-ECM adhesion complexes^7^.

**RGD (Arg-Gly-Asp tripeptide)** is an amino acid motif naturally found on extracellular matrix proteins. It is recognized by members of the integrin transmembrane protein family which is primarily responsible for the adhesion of cells to the ECM. Integrins are composed as the combination of two subunits, forming a total of 24 different types of proteins in higher vertebrates, eight of which are specific to RGD binding sites (αvβ3, αvβ5, αvβ6, αvβ1, αvβ8, α5β1, αIIbβ3, and α8β1)^8^. Novel chemical tools developed to functionalize in vitro surfaces allow for the examination of specific molecular interactions during surface adhesion, such as the RGD-mediated activation and clustering of integrins^9–14^. RGD antagonists binding integrin promise new cancer therapies, especially for angiogenesis inhibition^15,16^. RGD-based nanotherapeutics to target integrins on cancer cells are under development^17^, as integrins are extracellularly accessible. Preclinical trials provided promising results in various cancers.

The **eukaryotic cell cycle** is a series of biochemical events that leads to the duplication of chromosomes and their subsequent distribution to two daughter cells. The cycle, whose progression is controlled by members of the cyclin-dependent kinase (CDK) family in association with partner cyclin proteins^18^, affects most of the essential molecular machinery of the cell, including DNA and protein metabolism, growth, cytoskeletal rearrangements, and also integrin-mediated cell adhesion. The changing ratio of the CDKs and their counteractive phosphatases is thought to drive the progression of the cell cycle, as this ratio is sensed by substrates, which phosphorylate accordingly, propelling the transition through G1, S, and M phases^19^.

In cancer, there are fundamental alterations in the genetic control of cell division, resulting in an unrestrained cell proliferation^20^. During mitosis, microtubule dynamic is increased to 20–100-fold which suggests that mitosis is the primary time of microtubule targeting. Most microtubule targeting agents (MTAs) are also frequently referred to as “antimitotic drugs”^21^. MTAs can be divided into microtubule-destabilizing agents (such as the vinca alkaloids and colchicine), and microtubule-stabilizing agents (such as the taxanes)^21–24^, categories. The development of anticancer therapies targeting the microtubules has usually focused on agents that bind either the taxane or vinca alkaloid site^21^.

For most cells, cell cycle progression is anchorage-dependent^25^, requiring cell–ECM interactions via integrin transmembrane receptors and the formation of actin-associated adhesion complexes^26^. G1 and G2 phases are characterized by cell growth (intensive protein synthesis and organelle multiplication) which includes an increase in the area of contact between the cell and its substrate. Early observations on flat surfaces have shown that directly before mitosis (the M phase of the cell cycle), focal adhesion complexes and actin stress fibers are rapidly disassembled. This results in a drastic reduction of traction forces^27^ and previously spread out cells round up due to a radical reorganization of the cytoskeleton^28^. This cell rounding is required for accurate spindle formation and chromosome capture and the integrin-mediated adhesion is required for determining the orientation of cell division^29^. Focal adhesions provide essential cues to the anchorage-dependent cell cycle progression, and right before mitosis they undergo remodeling which allows the cell to round up. This decrease in adhesion activity is directly regulated by CDK1 through phosphorylation sites present on adhesion complexes^18^. Hyperactivation of integrins inhibits rounding up thus decreasing the ability of cells to divide. Cells regain the ability to produce traction forces as they exit from mitosis and re-spread onto the extracellular matrix in early G1^27^.

A few **methods** have been already suggested **to quantify the adhesion mechanics** exerted by the cells **throughout the cell cycle**. Table 1 summarizes and compares the relevant techniques available. An approach to examine this correlation is to use traction force microscopy (TFM)^30^ (Table 1). This technique measures traction forces indirectly by attaching cells onto an elastic surface containing fluorescent landmarks. As the cells exert mechanical forces on their substrate, the elastic gel including the markers deforms. Traction forces are derived from comparing the original position of the markers with their location after the cells have adhered and deformed the gel. An alternative way to measure traction forces is to use microneedles instead of fluorescent markers. In this setup, the needles designated for the cells to adhere onto are positioned in a discreet array. These pillars deform as cells exert forces on them, and their deformation serves as the basis for determining traction forces^31,32^.

**Table 1:** Comparative table to overview the capabilities of different cell adhesion measurement techniques^36^.

	Single-cell RWG	RWG	CCM*	FluidFM BOT	AFM	TFM
Throughput	1000 s of cells in situ	1000 s of cells in situ	100 s of cells/30 min	~ 20 cells/hour	1 cell/0.5–1 h	~ 50 cells/several hours
Force sensitivity	nN**	no	nN***	nN	[5,150] pN	pN-nN scale
Single-cell resolution	Yes	No	Yes	Yes	Yes	Yes
Temporal measurement	Yes	Yes	No	No	No	Yes
End-point measurement	Possible	Possible	Yes	Yes	Yes	Possible
Cell survival	Yes	Yes	Yes	Yes	No	Yes
References	^41,42^(present study)	^13,14,38,39,43–46^	^46,52,57–59^	^36,50–53^	^18,33,47^	^30–32,34^

RWG biosensors are generally used to measure thousands of cells simultaneously from the time of suspending the cells in the medium until completely adhered, novel RWGs even have single-cell resolution capabilities. An alternative measurement is also possible when completely adhered cells are measured for a short amount of time, as discussed in this study. The computer-controlled micropipette, FluidFM, and AFM are all capable of single-cell measurements, but the time needed to measure a cell varies greatly, as well as the scale of the force sensitivity. *CCM is an abbreviation for computer-controlled micropipette. **when calibrated using FluidFM BOT^41^. ***This value is based on numerical simulations on the lifting force of microbeads^60^, and on direct comparison with FluidFM BOT force data^53^.

The role of focal adhesions in the rounding up of cells during mitosis was investigated at different phases of the osteosarcoma cell cycle using atomic force microscopy (AFM)^33^ (see Table 1). In a study, the procession of cell cycle in HeLa cells was blocked and then released at various time points to obtain cells precisely in G1, S, and G2 phases. Cells were then fixed and stained to examine adhesion complexes and actin cytoskeleton. Evaluation of the data revealed that the adhesion area per cell gradually increased from early G1 to the end of the S phase, at which point it started to decline throughout the G2 phase^18^.

Cell cycle-dependent force transmission was investigated on HeLa Fucci2 cells using quantum-dot-based traction force microscopy. They found a linear increase of basal area upon progression from the M/G1 to the G1 phase and the cell surface increment continued in late S/G2. Forces also increased from the M/G1 phase to G1 and lasted through early S and a slight decrease was measured toward the end of the cell cycle, in the late S/G2 phase^34^. It was also observed that traction forces first increased from early G1 to late G1 and S phase and then dropped after the S phase until G2 in RPE1-Fucci cells on fibronectin surface. They confirmed that cells entering S phase produced significantly higher traction forces than cells in early G1 or late G2^30^.

The natural mobility of HeLa Fucci cells was also investigated to understand the mechanisms of cancer cell migration into the surrounding tissue, using a PDMS microfluidic device^35^. The study conducted by Ledvina et al. showed that HeLa Fucci cells migrated at an even rate while in the G1, S, and G2 phases of the cell cycle, while mitotic cells showed a decrease in migration speed^35^.

**Techniques to measure the force of cell adhesion** can be divided into two categories: population methods and single-cell approaches^36^. The simple washing assay, spinning disk method, and flow chambers rely on hydrodynamic shear flow removing cells from the surface. These techniques can investigate a population of cells; however, they do not enable single cell targeting.

An alternative to measuring cellular adhesion with high sensitivity and high temporal resolution is the application of evanescent field based optical biosensors^37,38^ (Table 1). Here, the biosensor signal is directly proportional to the cell-substratum contact area and also correlates with the strength of adhesion in real-time^38,39^. Importantly, photonic crystal biosensors^40^ and novel resonant waveguide gratings (RWG)^41,42^ have large enough spatial resolution to resolve individual cells. This is an important development since RWG sensors employed in the majority of prior publications average the signals of thousands of cells over a relatively large sensor area (4 mm^2^). Even so, the RWG technology was found to be useful to reveal molecular scale kinetic interactions in the cell-substratum contact zone^13,14,38,39,43,44^, and its signals were compared with Optical Waveguide Lightmode Spectroscopy (OWLS)^45^ and Computer Controlled Micropipette^46^ data.

To directly measure the adhesion force of single cells, cytodetachment with an AFM tip^47^ or micropipette aspiration^48,49^ can be applied. Both of them are very low throughput methods. Note, using a modified AFM applying vacuum on cells with a fluidic micro-channel (Fluidic Force Microscopy, FluidFM), the throughput is increased by a factor of 10 compared to conventional AFM^36,50–53^ (Table 1). The optical signal of a high spatial resolution RWG (Single-Cell RWG) was recently calibrated by our group to adhesion force and energy values using a robotic fluidic force microscopy setup (FluidFM BOT), making possible to resolve the kinetics of single-cell adhesion forces and energy of large cell populations^41^.

A computer-controlled micropipette can measure the adhesion force per unit area of individual cells with relatively high throughput (hundreds of cells in ~ 30 min^52^) especially when compared to AFM or FluidFM (Table 1). A unique feature of the micropipette-based method is that single cells can be isolated and further investigated by e.g., scRNA sequencing^54–56^ after the adhesion force measurement.

The **Fucci** (Fluorescent Ubiquitination-based Cell Cycle Indicator) system was developed by Sakaue-Sawano et al. as a tool to visualize the dynamics of cell cycle progression^61^. HeLa-Fucci cells periodically express two fluorescent protein tagged molecules namely Geminin fused with monomeric Azami green (mAG-hGem) and Cdt1 fused with monomeric Kusabira Orange (mKO2-hCdt1) the expression levels of which oscillate periodically during the cycle: mKO2-hCdt1 levels are high during G1, while mAG-hGem levels are high during the S/G2/M phases^61^. No fluorescence is emitted in transition from M to G1, whereas both red and green fluorescence signals are emitted in early S phase^62^ (Fig. 2C). This setup allowed us to monitor cell cycle progression over the course of adhesion measurements and categorize single-cell force data according to the cell cycle phase.

**Figure 1:**
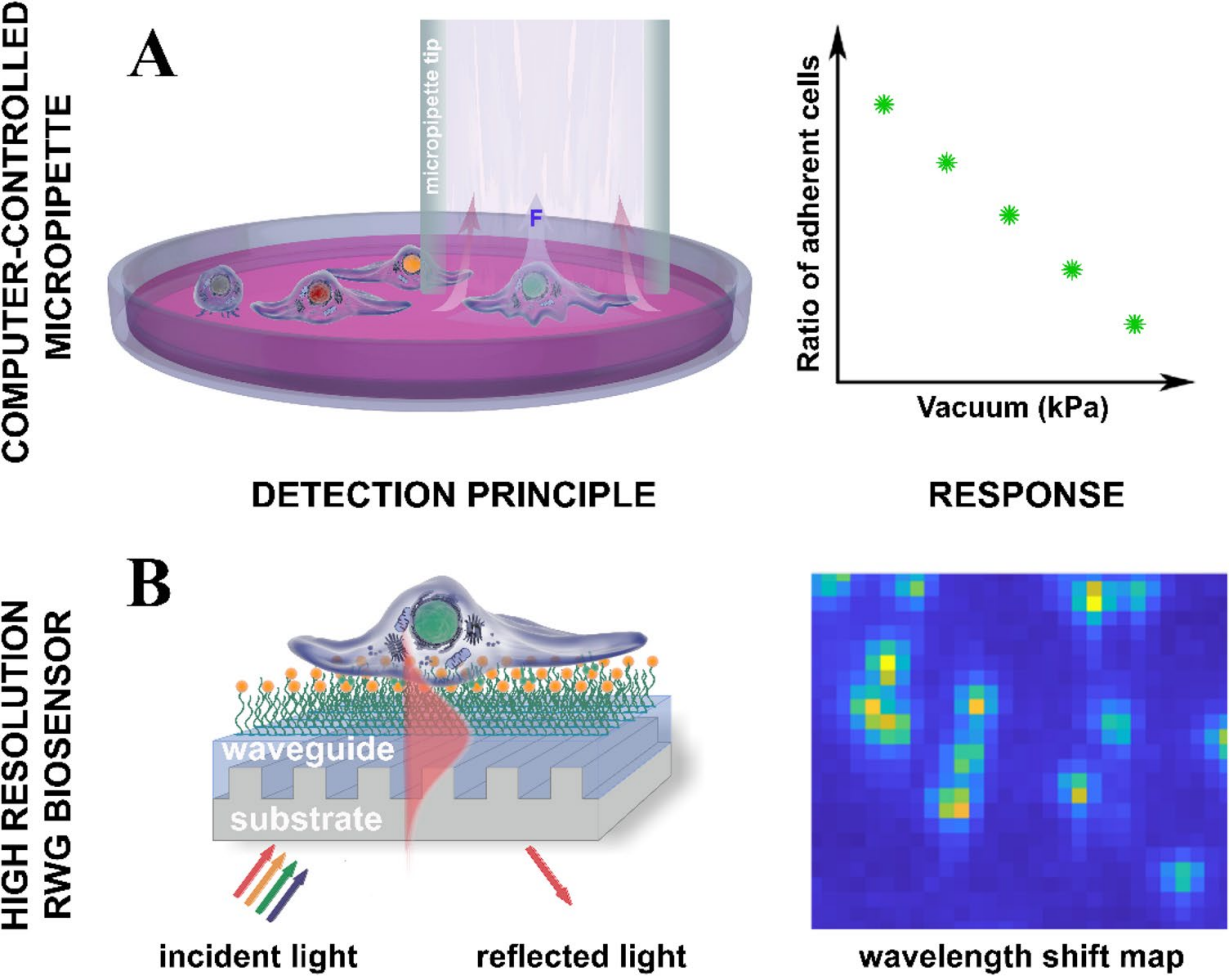
Schematic representation of the adhesion strength measurements on single cells using a computer-controlled micropipette^14^ and the high-resolution RWG biosensor (Single-Cell RWG)^39,40^. (**A**) Region of interest (ROI) of RGD-displaying Petri dish surface containing HeLa Fucci cells is scanned, then cells are automatically detected and selected for measurement. The developed device automatically adjusted the vacuum in a syringe connected to a micropipette with 70 micron opening, positioned the micropipette above the targeted cell and opened the fluidic valve. Adhesion characteristics of cells were evaluated by calculating the ratio of still adhering cells after the application of subsequent suction force steps on hundreds of cells. (**B**) Incident light is coupled into the biosensor chip through the waveguide grating and penetrates into a 150 nm depth into the sample (adhering cell) on the chip surface in the form of an evanescent field (red shadow above the waveguide surface). Thus field is ideal to monitor the cell-substratum contact zone. Biochemical or cellular events, such as cell adhesion change the local refractive index inside the evanescent field, resulting in a wavelength shift of the reflected light. Due to the large spatial resolution, individual cells are clearly visible on the recorded wavelength shift map. The wavelength shift is sensitive to nanometer scale changes in the cell adhesion contacts (perpendicular to the sensor surface). Such tiny variations are not resolvable by traditional optical microscopy.

**In our study,** we used cutting edge cell adhesion measurement techniques such as the computer-controlled micropipette^52^ and the previously mentioned single-cell resolution RWG biosensor (commonly recognized as Epic Cardio)^41,42^ to examine the dependence of cell detachment force on cell cycle progression (Fig. 1). To monitor the cell cycle phase of individual cells, we applied the Fucci genetic construct expressed by HeLa cells^61^. The computer-controlled micropipette can be mounted onto a standard inverted microscope. Cells are kept in a Petri dish and they are selected by software or the human operator based on their phase contrast and/or fluorescent images. Cells can be targeted individually by a glass micropipette with an aperture of 5–70 μm. Hundreds of cells adhered to specific macromolecules could be measured one by one in a relatively short period of time (~ 30 min). We could also measure the adhesion strength of several hundred cells in a label-free, non-invasive manner with a high temporal and lateral resolution in the RWG biosensor. The sensor itself consists of a transparent high refractive index waveguide layer with an incorporated grating. The primary signal of the sensor is the resonant wavelength shift (WS) of the incoupling light, which is given in picometers and is commonly referred to as dynamic mass redistribution (DMR) signal in the literature^63^.

## Materials and methods

### Cell culture for adhesion force measurement

HeLa Fucci (RCB2812, RIKEN BRC) cell line^61^ was maintained in Dulbecco’s Modified Eagle’s Medium (DMEM, 31,885 Gibco), supplemented with 10% fetal bovine serum (FBS) (Biowest SAS), 1% penicillin/streptomycin (Gibco) solution. Cells were cultured in a humidified atmosphere containing 5% CO_2_ at 37 °C.

### Single cell adhesion force measurement with computer-controlled micropipette

#### Cell preparation

The synthetic copolymer RGD-functionalized poly(L-lysine)-*graft*-poly(ethylene glycol) (PLL-*g*-PEG-RGD) was obtained as powders from SuSoS AG, Dübendorf, Switzerland and dissolved in 10 mM 4-(2-hydroxyethyl)-1-piperazine ethanesulfonic acid (HEPES, from Sigma-Aldrich Chemie GmbH, Munich, Germany) at pH 7.4. One day before measurement, 5 × 5 mm^2^ squares of the microwell in the Petri dish was coated with 100 µg/ml PLL-*g*-PEG-RGD at room temperature for 30 min. After that, PLL-*g*-PEG-RGD solution was removed from the well, and washed with 10 mM (4-(2-hydroxyethyl)-1-piperazineethanesulfonic acid) (HEPES) buffer 3 times. Then HeLa Fucci cells were removed from tissue culture dishes using trypsin–EDTA solution (Gibco, 25,300), and were seeded at 500 cells per well in 20 μl complete culture medium into the coated microwell and incubated overnight in a humidified incubator. Just before the sorting, the PDMS insert was removed from the Petri dish, and the whole of the Petri dish was flooded with cell culture medium.

#### Preparation of PDMS microwells

We molded 4-well inserts with four 5 × 5 mm^2^ wells from polydimethylsiloxane (PDMS, Dow Corning Sylgard 184)^57^. We attached the PDMS inserts with a height of 1 mm onto the bottom of a hydrophilic 35 mm Petri dish (Greiner Bio-One).

### Computer-controlled micropipette

Computer-controlled micropipette (CellSorter)^58,59^ was used to measure the adhesion of single cells on a microscope^52,53^ First, we inserted a glass micropipette with an inner diameter (ID) of 70 µm into the micropipette holder^57^ and filled it with deionized water. Then the I.D. 1 mm PTFE tubes^52^ were also filled with deionized water.

#### Image scanning

Cells in a Petri dish were placed onto a sample holder insert (CellSorter) fitting into the 2D motorized stage (Scan IM 120 × 100 motorized stage, Märzhäuser) of the inverted fluorescent microscope (Zeiss Axio Observer A1). We scanned the ROI by capturing a mosaic image covering the whole or a part of the 5 × 5 mm^2^ square in phase contrast and fluorescent modes. We used the micropipette head with rings of LED-s arranged concentrically to the micropipette for high quality phase contrast illumination of the sample^57^. Phase contrast images of the culture were captured by a digital camera (Andor Zyla sCMOS).

#### Cell detection

**Figure 2:**
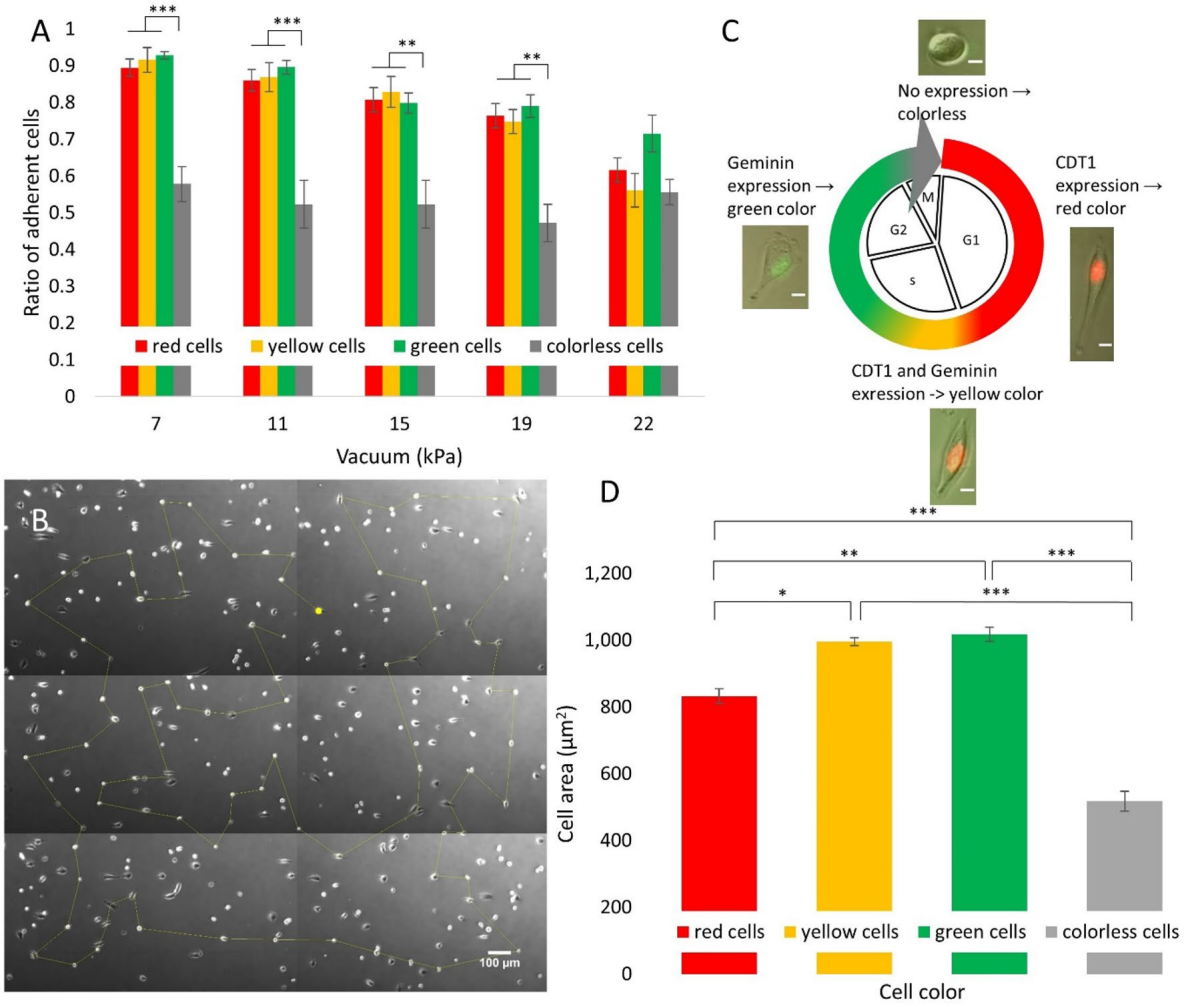
(**A**) Ratio of adherent HeLa Fucci cells on PLL-*g*-PEG-RGD surface at different vacuum values, as measured by the computer-controlled micropipette. * indicates significant difference between the ratio of different cell phases. (**B**) Phase contrast image of a small area of the Petri dish. The shortest path along all cells selected for measurement was calculated by the CellSorter software and projected onto the image in yellow. (**C**) Expression of the Fucci fluorescent marker set along the cell cycle. The red reporter is attached to the CDT1 protein, expressed early in the cell cycle, while the green reporter is attached to Geminin protein, which is expressed later in the cell cycle. Both reporters are expressed for a short time resulting in yellow color. Neither proteins are expressed during mitosis, making the cells invisible (colorless) in fluorescent images. Scale bar represents 100 µm. (**D**) Correlation between the cell color and the average cell area measured. Bar chart results are from 4 different micropipette experiments, where n = 88 for red cells, n = 90 for green cells, n = 95 for yellow cells and n = 32 for colorless cells. * indicates significant difference between the areas of cells of different colors.

Cells imaged in a phase contrast mode were automatically detected by the CellSorter software. The detection parameters (including sensitivity, cell brightness range and cell size) were manually tuned to optimize cell selection. To avoid picking up cells very close to each other we excluded cells when having neighbors in the 50–100 µm proximity. The shortest path of the micropipette visiting all cells was also determined by a travelling salesman algorithm (Fig. 2B). After cell detection, vertical position of the micropipette was precisely calibrated, using a motorized micromanipulator (Micromanipulator SM325, Märzhäuser). Height of the micropipette above the Petri dish was adjusted to 10 µm with ~ 1 µm positioning accuracy.

**Vacuum** in the range of [0, 22] kPa was generated in the syringe using a syringe pump. After positioning the micropipette above a cell, the valve was opened for 20 ms. (Pick up parameters: Valve1: 20 ms, Valve2: 0 ms, Delay: 0 ms). After each cycle of the adhesion force measurement, the ROI of the Petri dish was scanned again, and the vacuum was increased to the next level. Subsequently each detected cell was probed again by the micropipette. Suction force was increased until most of the cells were removed.

#### Ratio of adherent cells

The software determined the coordinates of the cells by computer vision and saved them before and after each cycle of the adhesion force measurement. The cell coordinates before and after the cycles were compared to calculate the ratio of still adherent cells to the cell number placed onto the surface at the beginning of the experiment.

#### Determination of the threshold fluorescence of cells

Expression level of the two fluorescent reporter proteins changes continuously throughout the cell cycle. To define the four categories for the four states of red/yellow/green/colorless cells, a threshold fluorescence intensity value must be determined in both channels. We measured the corrected total cell fluorescence (CTCF) of cells that were slightly above our human visual detection threshold with ImageJ^64^. (CTCF = Integrated pixel brightness of the cell – Area of selected cell X Mean brightness of the background of selected cell.) Then we normalized the CTCF value by the cell area. We found that the threshold value of CTCF/(cell area) was 4.28 ± 0.42 in case of green cells and 5.37 ± 0.42 in case of red cells, respectively. The employed threshold CTCF/cell area value was 4.3 and 5.4 in the green and red channels, respectively.

#### Determination of the cell areas

Cell width (a) and height (b) on images were measured with the ImageJ software^65^. Cell area (A) was calculated for n = 87 red cells, n = 90 green cells, n = 91 yellow cells, n = 40 colorless cells from these parameters by approximating cell shape as an ellipse:

1 $$A=\frac{a}{2}*\frac{b}{2}*\pi$$

### Single cell adhesion strength measurement with high spatial resolution resonant waveguide grating optical biosensor (Single-Cell RWG)

The Epic Cardio RWG imager biosensor^41^ (Corning Inc., Corning, NY, USA) was used in our experiments which accepts 384-well Society for Biomolecular Screening (SBS) standard format biosensor microplates. The plate is illuminated from below and the light is coupled into the thin high refractive index (RI) waveguide layer^41^. The light propagation happens here through a series of total internal reflections in the form of a waveguide mode (illustrated as the red shadow in Fig. 1B.) The sensing principle is based on a phase shift that occurs during total internal reflection events, which is dependent on the refractive index of the close vicinity of the surface of the waveguide. Here, changes in the local refractive index are monitored by an optical evanescent field penetrating into the adhering cells to a depth of around 150 nm. A dedicated optics with a complementary metal-oxide semiconductor (CMOS) camera is used to detect the outcoupled light and its WS map (see Fig. 1B). The camera used provides a final biosensor image spatial resolution of 25 µm and a sampling time of 3 s. Note, dead cells are excluded from the analysis, their signal is negligible in waveguide sensing^66^.

#### Cell preparation

A standard, 384-well biosensor microplate was coated with the synthetic polymer PLL-*g*-PEG-RGD solution as detailed in the former ‘*Cell preparation*’ section. After the wells were filled up with the solution, the microplate was centrifuged (Allegra X-30R, Beckman Coulter) at 300×*g* for 10 s to sink the solution to the bottom of the wells, and then the microplate was swayed for 30 min to achieve an equal distribution of the coating solution. After that, the solution was removed, the wells were washed with 10 mM HEPES buffer 3 times. HeLa Fucci cells were detached from tissue culture dishes using trypsin–EDTA solution and then the cancer cells in four different concentrations (300, 200, 100, and 50 cells per well) were pipetted into the custom well containing the array of 2 × 2 mm optical sensors. Cells were incubated in the Epic microplate overnight at 37 °C and 5% CO_2_ atmosphere.

#### Biosensor measurement

Following the overnight incubation, the microplate was placed into the high-resolution RWG imager and the wavelength shift map of the adhered individual cells was measured for ~ 10 min at room temperature.

#### Cell detection using microscopy

After biosensor measurements, the biosensor microplate was removed from the RWG imager and inserted into a fluorescent microscope (Zeiss Axio Observer Z1). We captured phase contrast and fluorescent mosaic images by a digital camera (Blackfly S, Flir). Fluorescent and phase contrast images were combined in ImageJ software^65^ to represent the fluorescent characteristics of the cells, that is, their phase in the cell cycle, based on the color of their nucleus.

#### Statistical analysis

Student’s t-test (unpaired, two-tailed, unequal variance) or two-way ANOVA/Turkey’s multiple comparison test was used to determine significant differences between our results. Statistical significance is denoted on our graphs by * for P < 0.05, ** for P < 0.01, *** for P < 0.001 and **** for P < 0.0001. Bar graphs showcase the mean of experimental data ± SEM. Data were analyzed and visualized using Microsoft Excel software. Statistical testing for significance was done by GraphPad Prism 6.0 (GraphPad Software, La Jolla, CA, USA).

## Results

### Single cell adhesion strength measurement with computer-controlled micropipette

#### Determination of cell color (cell phase) during the cell cycle, based on microscope images

Fluorescent and phase contrast images of the ROI were captured by the CellSorter software which automatically recognized the cells in the images and calculated their coordinates. Comparison of cell coordinates in multiple channels allowed the characterization of cells into four groups: ‘red cells’: cells that appeared only in images captured in the red fluorescent channel due to the presence of the red fluorescent marker; ‘yellow cells’: cells that appeared in both fluorescent channels due to the presence of both red and green fluorescent markers, ‘green cells': cells appeared only in the green fluorescent channel, and ‘colorless cells’: cells that did not appear in any fluorescent channel. Fluorescent and phase contrast images were combined to provide a visual representation of the cells we investigated.

#### Adhesion characteristics of HeLa Fucci cells

**Figure 3:**
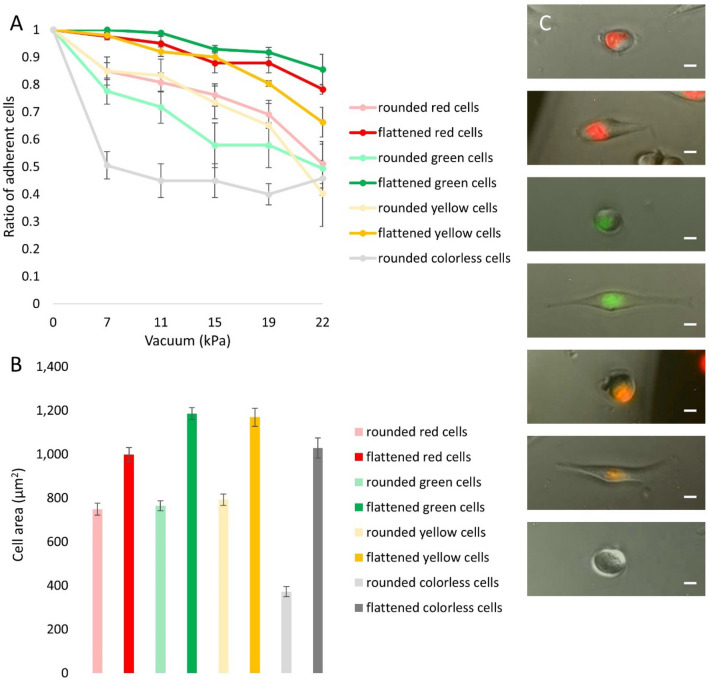
(**A**) Cell adhesion strength of HeLa Fucci cells on PLL-*g*-PEG-RGD surface as a function of cell color and morphology measured with the computer-controlled micropipette. Aggregated cell adhesion data shown in Fig. 2A were separated on the basis of cell morphology. As expected, round cells showed a lower affinity to adhere to the surface than flat cells did, regardless of cell color. (Flattened colorless cells were very rare, thus we could not collect enough data to obtain their adhesion curve.) (**B**) Determination of the average cell area as a function of cell color and cell morphology. All charts show results from 4 different micropipette experiments, and n = 31 flattened red cells, n = 57 rounded red cells, n = 56 flattened green cells, n = 34 rounded green cells, n = 57 flattened yellow cells, n = 38 rounded yellow cells, n = 32 rounded colorless cells**.** (**C**) Cells were divided into eight groups based on their color and morphology. From top to bottom: rounded red cell, flattened red cell, rounded green cell, flattened green cell, rounded yellow cell, flattened yellow cell, rounded colorless cell. Scale bars represent 10 µm.

We found that red, yellow, and green cells showed similar adhesion characteristics, while we observed a significantly lower resistance against the hydrodynamic lifting force in the case of colorless cells, measured with the micropipette (Fig. 2A). Besides color, cells were further categorized into two groups based on morphology. We classified rounded cells with no visible protrusions into the ‘rounded’ category, while spread out cells with protrusions were classified into the ‘flattened’ category (Fig. 3C). We observed that flattened cells adhered significantly stronger to the coated surface during the three smallest vacuum value than rounded cells did (rounded cells: 0.74 ± 0.05, flattened cells: 0.98 ± 0.01* at 7.07 kPa; rounded cells: 0.70 ± 0.05, flattened cells: 0.96 ± 0.01** at 11.26 kPa; rounded cells: 0.63 ± 0.08, flattened cells: 0.92 ± 0.02* at 15.09 kPa). Cell adhesion as a function of cell color and morphology was summarized to examine the correlations. As expected, rounded cells showed a lower adhesion strength than flattened cells did, regardless of cell color (Fig. 3A).

#### Determination of the cell area

We found that green cells had the largest, and colorless cells had the smallest cell area (Fig. 2D), which correlates with the adhesion strength in Fig. 2A.

We examined the cell area as a function of cell morphology and we found that flattened cells had a much larger area than rounded cells (Fig. 3B).

### Single-cell adhesion force measurement with the high spatial resolution RWG biosensor

Optical biosensors were previously successfully applied in real-time monitoring of eukaryotic cell adhesion as presented earlier^41^. As the instrument’s sensing volume overlaps with the adhered cell’s adhesion zone it inherently captures cell mass redistributions related to adhesion molecule binding and/or cytoskeletal reorganization^38^. It is important to note, the recorded wavelength shift is sensitive to nanometer scale changes in the cell adhesion contacts (perpendicular to the sensor chip surface), to such tiny variations which are not resolvable by traditional microscopy.

#### Identification of cells on the biosensor microplate

**Figure 4:**
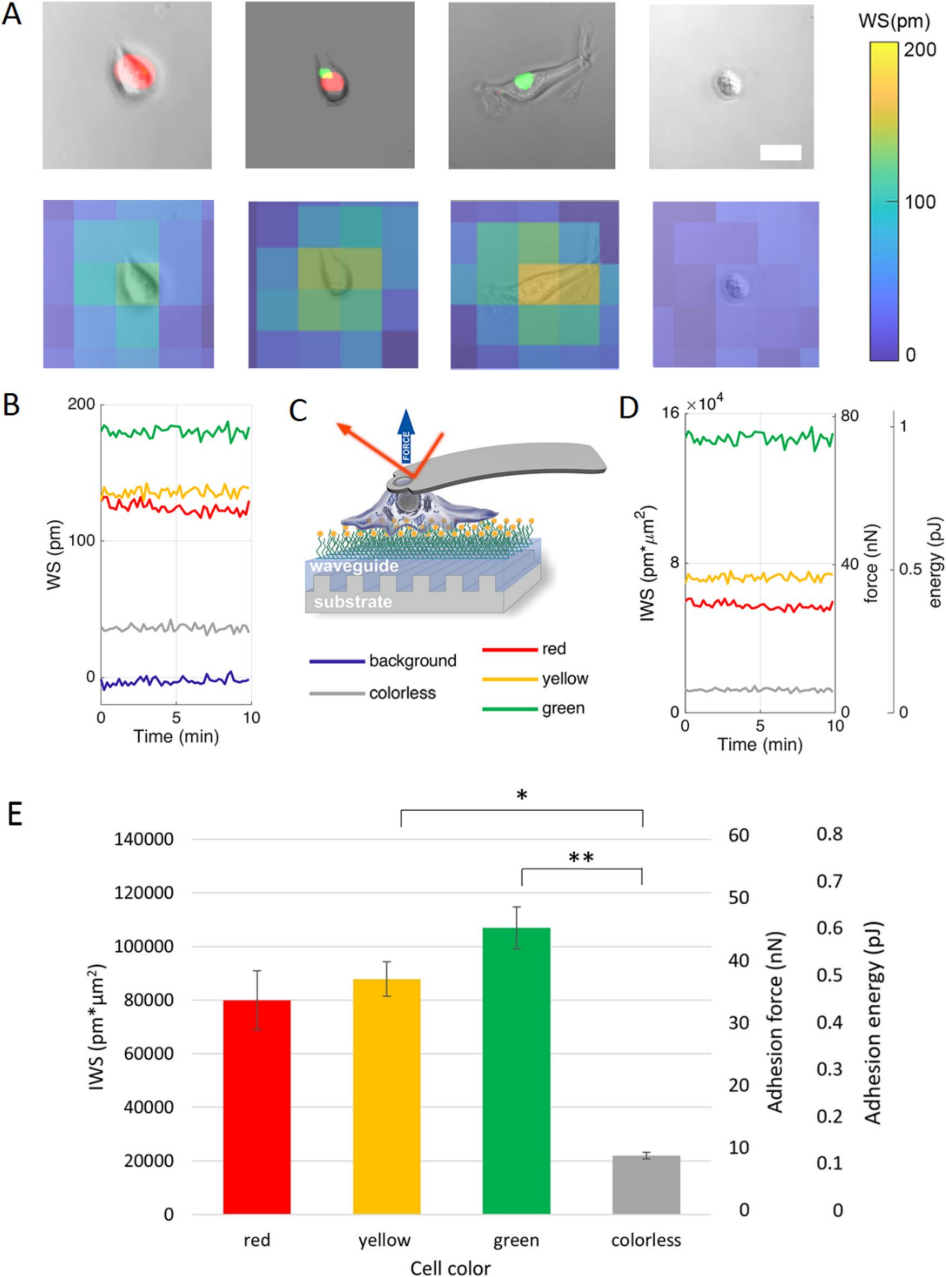
Comprehensive representation of the biosensor data. (**A**) Top row: Phase contrast and fluorescent composite images of typical red, yellow, green, and colorless cells (respectively). Scale bar represents 25 µm. Bottom row: Composite images of the respective top row cells overlayed from the biosensor wavelength shift map and the phase contrast image at t = 10 min. (**B**) Near-stationary time dependence of maximal biosensor pixel values (WS) for representative cells depicted in Fig. 4A. (**C**) Schematic representation of the robotic fluidic force microscopy (FluidFM BOT) measurement process. A special hollow FluidFM cantilever capable of whole cell force-spectroscopy was used to calibrate biosensor signals for adhesion force and energy conversion as described in^41^. (**D**) Time-dependence of IWS and deduced force/energy signals of single cells in different phases of the cell cycle. (**E**) Correlation between cell color and IWS, adhesion energy, and adhesion strength. A significant difference was found between colorless cells with respect to green and yellow cells. Graphs show the average of n = 7 red cells, n = 11 green cells, n = 8 colorless cells, and n = 11 yellow cells ± SEM.

Phase contrast and fluorescent images of the microplate wells were overlaid with ImageJ (Fig. 4A top row) to determine the color of cells and be categorized into the groups specified in the ‘*Determination of cell color (cell phase) in the cell cycle, based on the microscopic images’* section. After that, the biosensor image (wavelength shift map) of the microplate wells was projected onto the overlaid phase contrast and fluorescent images to allow easy assignment of biosensor signal to an actual cell and its color (phase in the cell cycle) (Fig. 4A bottom row). Moreover, the image created this way aided to select cells suitable for evaluation, i. e. cells of healthy morphology that had no neighbors in their close vicinity. Exception from this criterion were the cells in M-phase (colorless cells), that were usually found in pairs close to each other after division.

#### Biosensor signal processing and cell color

Adherent single HeLa Fucci cells show a distinct WS peak signal emerging from the background on the 2-dimensional wavelength shift map (Fig. 4B). Our previous studies showed a linear relationship between the surface integral of WS (IWS) and standard biophysical measures such as single cell detachment force and energy^41^, thus besides raw WS values, we calculated this variable for each time step as well (Fig. 4C). Since typical cell size was larger or in the range of the spatial resolution of the instrument, IWS was defined as the product of maximal WS pixel value corresponding to a given cell and the cell area measured on the microscopic images. As shown in Fig. 4 raw WS data—and the derived IWS—could be considered stationary on the timescale of measurement, thus it gives a good basis for comparison of adhesion states.

After identification of the cells on the biosensor (Fig. 4A), wavelength signals were categorized into the 4 groups provided by cell color. n = 11 single green cells, n = 7 single red cells, n = 8 single colorless and n = 11 single yellow cells for determination of WS and IWS were identified on the overlaid biosensor and microscopic image. The comparison of the identified subpopulations is shown in Fig. 4E. We found that colorless cells had significantly lower IWS signals – corresponding to lower overall adhesion force and energy values than yellow and green cells, and no significant difference could be found between the IWS values of red and colorless cells. (Fig. 4E).

**Figure 5:**
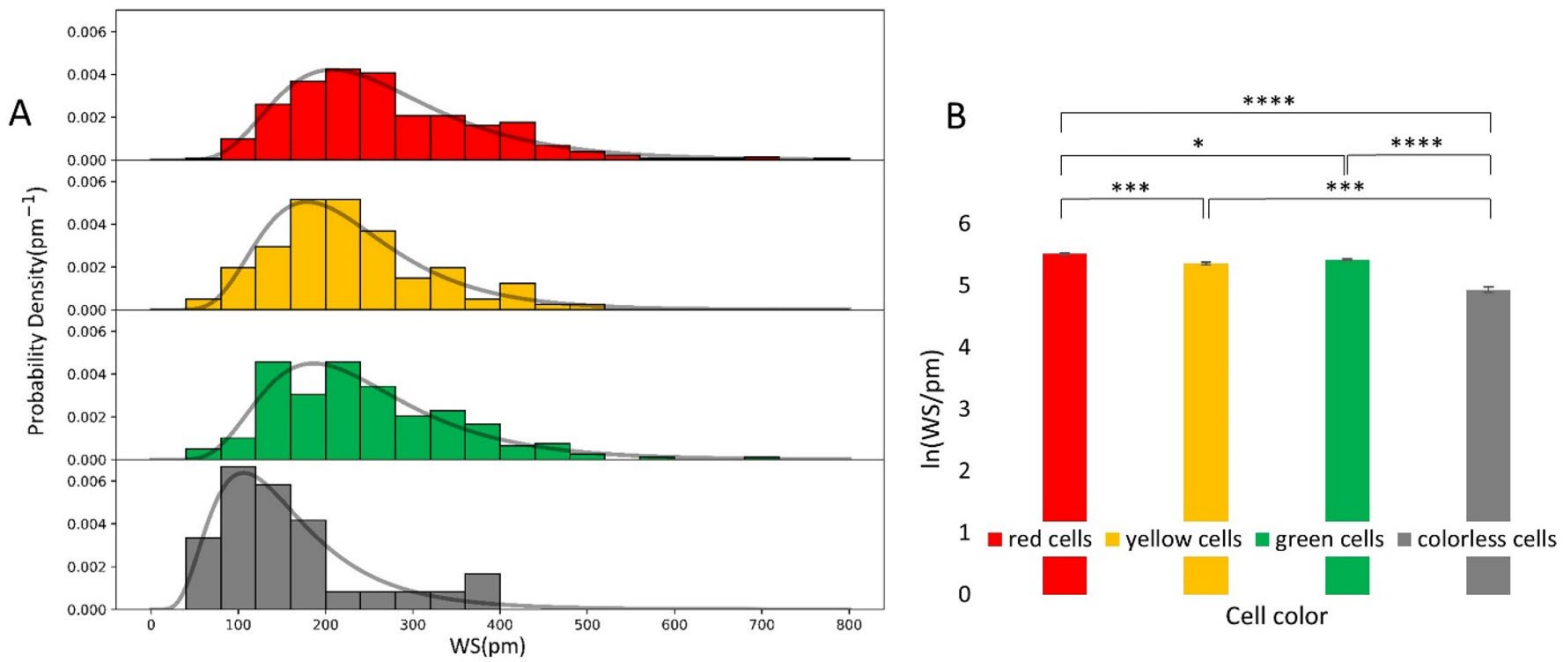
Biosensor data of the recorded single-cell signals at a single pixel underneath of each cell. (**A**) The local biosensor signals of individual cells (maximal WS pixel value corresponding to a given cell) followed a lognormal distribution (solid line) in all cell-cycle states. (**B**) Normalized data showed significant differences between the various phases in a decreasing order of red, green, and yellow cells, with a distinctly less signal for the colorless cells. Mean ± SEM is shown. (n = 325 red cells, n = 197 green cells, n = 30 colorless cells, and n = 102 yellow cells).

In order to better use the unique capabilities of the biosensor, we also analyzed the single pixel values of the recorded data. The maximal WS pixel values corresponding to the individual cells were recorded for a relatively large population of cells in all color states, and the population distribution of the signals were analyzed. It is important to note, a single pixel WS value is proportional to the cell mass per unit area inside the cell adhesion contact zone. This quantity is defined as contact mass density or simply contact density in the present work. Of note, 100 pm WS signal roughly corresponds to 300 pg/mm^2^ surface mass density^45^. The recorded distributions were found to be lognormal in all states (see Fig. 5A). It is revealing that WS values above 400 pm are completely missing for the colorless population and this sub-population have a clearly less distribution mode value than that of the colored populations (see Fig. 5A). The differences with statistical analysis are further highlighted in Fig. 5B. These results suggest that the cell-substratum contact zone has a remarkably different composition or/and morphology in the mitotic phase, the measured weaker overall single-cell adhesion is not simply a direct consequence of the recorded smaller adhesion area (see Figs. 2, 4).

## Discussion

We investigated and compared cell adhesion strengths throughout the cell cycle at the single-cell level using two complementary experimental methods: computer-controlled micropipette and a high spatial resolution RWG optical biosensor. Our goal was to determine how the strength of adhesion of cells to the surface (extracellular matrix) varies during the progression of the cell cycle. To mimic the in vivo environment, we used a synthetic polymer, PLL-*g*-PEG-RGD surface. As a cell cycle reporter, we used the Fucci fluorescent construct in HeLa cells. Fucci expresses two different fluorescent reporter proteins in a time-varying quantity during the cell cycle. Observation of the cell culture on a fluorescent microscope allowed us to categorize cells into four groups based on their fluorescent intensity: red cells (G1 phase), yellow cells (beginning of the S phase), green cells (S/G2/M phase), and colorless cells (transition from M to G1 phase).

The combination of optical biosensor measurements and the computer-controlled micropipette allowed us to study the adhesion strength throughout the cell cycle in a more detailed way than ever before, particularly, during and around cell division. The computer-controlled micropipette applied a stepwise increasing suction force on cells to pick up the targeted cell from the RGD-coated surface. This technique probes the adhesion strength of cells per unit area. The ratio of still adherent cells after the application of each level of suction force revealed that colorless cells needed a much lower hydrodynamic lifting force to be picked up from the surface than the colored cells did. No significant difference could be found between the adhesion strength of red, yellow, and green cells. Label-free biosensor measurements confirmed the weaker adhesion of colorless cells showing a much lower biosensor signal. Comparison of red, yellow, and green cells revealed no significant difference in the sensor signal. Maximum wavelength shift represents the averaged adhesion strength over a physical pixel (25 × 25 microns) of the imager biosensor. Since the computer-controlled micropipette data is in connection with the surface averaged adhesion strength over the area of a given cell, the results of the two different techniques are perfectly in line. It can be safely concluded that HeLa Fucci cells strengthen their adherence after mitosis, and maintain their strong adhesion throughout the cell cycle, decreasing it only once the cell gets ready for division in the late M phase. As our proposed method allows real-time monitoring of adhesion energy and force as well via conversion of the single-cell IWS signals, we could also conclude that these measures show similar dynamics throughout the cell cycle. Of note, we observed lower WS values and a lower adhesion strength with HeLa Fucci cells than in our previous study with non-transfected HeLa cells^41^. Our results thus are directly comparable with other works in the literature on single-cell force spectroscopy, with the prominent advantage over them, being a non-invasive, label-free, and real-time method. Moreover, the developed methodologies are ready to be employed in more complex lab on a chip system for industrial applications, to test the effect of novel drug candidates on cancer cells during their cell cycle progression.

Our observed dynamics are in agreement with the results of Panagiotakopoulou et al.^34^ using quantum-dot-based traction force microscopy of HeLa Fucci2 cells. They also reported significant weaker total traction force in M and early G1 phases (colorless cells) than in late G1 throughout G2 (colored cells). They found that not only the overall force exerted by the cells was significantly lower right after the M phase but the traction force normalized by the area, as well. Therefore, the traction force is expected to be actively downregulated in the late M phase. Another study by Vianay et al.^30^ observed a significant increase in normalized traction energy between early G1 and the S phase in RPE1-Fucci cells. In Refs.^30,34^ they found a significant difference between yellow and green cells but this difference did not appear in our measurements.

The main characteristic of cell cycle progression is cell growth: cell size and mass increase steadily from early G1 to late G2^67^. We found that colorless cells (late M, early G1) had the smallest area on the adhesion surface. Red and yellow cells (G1 and beginning of S) had a larger area, and green cells (S/G2/M) were the largest.

From the measurements on adhesion strength we found that the overall adhesion strength of single cells is constant in all phases, except the mitotic (M) phase with a significantly lower adhesion. It is important to note that, cells adhere during the M phase in a specific, atypical way, using the so-called reticular adhesions, which are very distinct from canonical focal adhesions^68^. Our results perfectly support this finding and highlight some additional differences. By employing a high spatial resolution RWG optical biosensor we could measure the cell mass per unit area inside the adhesion contacts during the cell cycle progression. We found that the adhesion contact is significantly less dense during the mitotic phase, suggesting a distinct change in adhesion contact morphology or/and composition. Therefore, our results suggest that the weaker mitotic adhesion is not simply a direct consequence of the measured smaller contact area in the M phase.

Cell adhesion features already play an important role in various cancer treatments. Recently Kubiak et al.^69^ demonstrated that treatment with microtubule-targeting antimitotic drugs, such as vinca alkaloids and taxanes, can change actin cytoskeletal organization and cell mechanics, influencing drug resistance. Another study highlighted that integrin-mediated cell adhesion can have an impact on cancer drug sensitivity^70^. Moreover, drug-resistant cancer cells (towards MAPK inhibitors, which also affect the cell cycle) are characterized by cytoskeletal remodeling, high myosin II and Rho activity. Our finding suggests that cell adhesion is a promising target of selective cancer drugs, as the vast majority of normal tissue cells do not enter the M phase. We believe that cell adhesion may be a new tumor therapeutic target^16,17^ because its strength is significantly reduced at the end of the M phase. Since this is a short period of the cell cycle, the effect of a drug can be specific to the rapidly dividing tumor cells. Given the wide clinical use of vinca alkaloids and taxanes, currently, the microtubules represent the best target to date for cancer chemotherapy^69^. However, several clinical trials have proved integrins to be a promising therapeutic target to battle cancer cells, though no specific drug was found to be exceptionally effective^71^. We believe the experimental techniques used in our study and the results provided by them to be of aid to develop novel integrin targeting drugs.
